# Eficacia del método de vahanwala en la determinación del sexo a través de la queiloscopia en impresiones escaneadas de un grupo de estudiantes peruanos

**DOI:** 10.21142/2523-2754-0903-2021-067

**Published:** 2021-10-06

**Authors:** Lesly Yuliana García Jáuregui

**Affiliations:** 1 División de Odontología Legal y Forense, Universidad Científica del Sur. Lima, Perú. lgraciajauregui88@gmail.com Universidad Científica del Sur División de Odontología Legal y Forense Universidad Científica del Sur Lima Peru lgraciajauregui88@gmail.com

**Keywords:** método de identificación odontológica, impresiones de los labios, certeza del sexo, Cheiloscopy, lip prints, sex determination

## Abstract

**Objetivo:**

Demostrar la eficacia para determinar el sexo mediante la queiloscopia utilizando el método de Vahanwala en impresiones labiales escaneadas en la Institución Educativa de Menores Túpac Amaru Apurímac 2017 (Lima, Perú). Para el estudio se recogieron 94 muestras huellas labiales, las cuales 44 fueron de varones y 50, de mujeres. Las huellas labiales se realizaron utilizando lápiz labial color negro, cinta adhesiva, hisopos y papel bond. Posterior a la recolección se utilizó el escáner HP Deskjet 2540, con el cual se obtuvieron las imágenes para ser analizadas con el programa Adobe Photoshop CS6. Se registraron y evaluaron los siguientes ítems; tipos de surcos labiales, distribución de las comisuras labiales, tipo de grosor labial y la relación que poseían en la diferenciación del género. La concordancia entre el resultado del método y la condición real del sexo fueron evaluadas con la prueba estadística de Kappa.

**Resultados:**

El 55,4% presentó comisuras labiales de tipo horizontal para ambos sexos, con relación al grosor labial; el 45,5 % del sexo masculino presentó labios de tipo medianos, mientras que el 34,0% del sexo femenino presentó labios de tipo mixtos, en relación con los surcos labiales analizados en la impresión labial escaneada. Para los hombres hubo predomino de la clase IV (59,2%) y para las mujeres la clase I y II (72,0%). En cuanto a la clasificación precisa del método Vahanwala empleado en el estudio, existe exactitud para la determinación del sexo utilizando impresiones labiales escaneadas.

**Conclusiones:**

El método de Vahanwala es eficaz para la determinación del sexo utilizando impresiones labiales escaneadas.

## SUMMARY

**Objective:**

To demonstrate the effectiveness to determine sex through cheiloscopy using the Vahanwala method in scanned lip impressions at the Tupac Amaru Apurímac Secondary Training Entity for Minors in Lima, Peru in 2017.

**Methods:**

94 lip imprint samples were collected for the study, of which 44 were from male and 50 from females. The lip prints were made using black lipstick, adhesive tape, swabs, and bond paper. After the collection, a HP Deskjet 2540 scanner was used to obtain images of the lip prints, and these were analyzed with the Adobe Photoshop CS6 program. The following aspects were recorded and evaluated; type of labial grooves, distribution of the labial commissures, lip thickness and the relationship these aspects had in the differentiation of gender. Concordance between the result of the method and the real sex of the lip print was evaluated with the statistical-kappa test.

**Results:**

55.4% presented horizontal labial commissures in both sexes, 45.5% of the males presented medium lip thickness, while 34.0% of the females presented mixed type lip thickness. In relation to lip furrows, class IV furrows predominated in 59.2% of men, with class I and II furrows being found in 72.0% of women.

**Conclusions:**

The Vahanwala method is effective for sex determination using scanned lip impressions.

## INTRODUCCIÓN

La palabra *queiloscopia* proviene de los términos griegos *cheilos* y *skopía*, y significa ‘estudio de las impresiones de los labios’. Es definido como el procedimiento de identificación basado en la disposición de los surcos que aparecen en los labios, o como una ciencia que estudia los surcos que aparecen en los labios. Las impresiones labiales son similares a las huellas dactilares, palmares y las huellas que se utilizan para la identificación individual. Fischer fue el primer antropólogo en describir los surcos labiales humanos, en 1902. El uso de huellas labiales se recomienda tan pronto como en 1932, por Edmond Locard, uno de los más grandes criminólogos de Francia. LeMoyne Snyder realizó una investigación de homicidios, publicada 1950, en la cual se menciona la utilización de las huellas de los labios para determinar la identidad de las personas. En 1967, Santos fue la primera persona que clasificó los surcos labiales en tipos simples y compuestos. El tipo simple se subdivide en cuatro tipos: línea recta, línea curva, línea en ángulo, y línea curva con forma sinusoidal. El tipo compuesto se subdivide en grupos bifurcados, trifurcados y anómalos. En 1981, Cottone informó que la queiloscopia es una de las técnicas especiales que se utilizan con el propósito de identificación ^1^^-^^8^.

Numerosos investigadores analizaron el posible uso de las impresiones de los labios para elaborar certificados de identificación en las personas. En 1967, Suzuki y Tsuchihashi efectuaron un estudio con la cual obtuvieron muestras de huellas labiales en varios sujetos para aplicar y aumentar los estudios de peritos, lo que perfeccionó dicha práctica. Investigaciones posteriores realizadas por Renaud, en los años 70, ratificaron la singularidad de cada huella labial ^9^^,^^10^.

Existen variadas clasificaciones, elaboradas por numerosos autores, que se han unido al estudio de la queiloscopia. Entre las más destacadas se encuentran las de Santos, Renaud, Kasprzak, Afchar-Bayar y Suzuki y Tsuchihashi. En discrepancia con los demás investigadores, Suzuki y Tsuchihashi clasificaron las impresiones labiales en torno a la forma y el recorrido de las ranuras en seis clases. A partir de todas las investigaciones que se realizaron para la clasificación de tipos de surcos labiales, Vahanwala *et al*. propusieron 4 patrones de clasificación para la determinación del sexo ^10^^,^^11^.

En la actualidad, se hacen necesarios más trabajos de queiloscopia que consoliden los resultados encontrados en otros grupos raciales y países. En el Perú, existen pocas investigaciones al respecto; por tal motivo, el presente trabajo busca demostrar la eficacia para determinar el sexo mediante la queiloscopia utilizando el método de Vahanwala, en impresiones labiales escaneadas en la Institución Educativa Túpac Amaru, Apurímac, en 2017 (Lima, Perú).

## MATERIALES Y MÉTODOS

Ese estudio transversal evaluó 94 muestras de impresiones labiales estudiantes matriculados en el año 2017 en la Institución Educativa Túpac Amaru, del departamento de Apurímac, provincia y distrito de Chincheros (Lima, Perú). Los criterios de exclusión que se establecieron antes de iniciar la investigación fueron los siguientes: patologías orales en los labios, secuelas de las patologías orales en los labios, malformaciones de tipo genético o adquirido, y pigmentación labial.

### Selección de la muestra 

Para realizar la selección de la muestra, se incluyó al grupo de estudiantes que contaban con el asentamiento y consentimiento informado. A cada participante se le explicó el objetivo de la investigación.

### Recolección de la muestra

Para el inicio de la recolección de la muestra, inicialmente, se procedió a la calibración del programa Adobe Photoshop 7.0, previa instalación en la laptop Toshiba Intel Core i5, mediante el cual se visualizarán las huellas labiales escaneadas.

La disposición de las comisuras labiales se obtuvo con la observación directa e indirecta del labio (fotografía digital), al igual que el grosor de labio.

Se les pidió a los participantes del estudio que mojen sus labios para, posteriormente, limpiarlos con un algodón húmedo, y se dejó que se sequen durante un minuto. Con un lápiz labial de color negro, se realizó la aplicación de manera uniforme en el bermellón de los labios. Se les pidió a los participantes del estudio que froten los labios poco a poco, con el objetivo de expandir el lápiz de labios uniformemente por todo el labio. 

Después de dos minutos de la aplicación del lápiz de labios, se procedió a pegar la cinta celofán en los labios utilizando la técnica de derecha a izquierda logrando, de modo que queda colocada de manera uniforme y sin ninguna burbuja. La cinta cubrió ambos labios, sin ningún movimiento. La tira de celofán se retiró utilizando la misma técnica que de izquierda a derecha, para ser pegado correctamente, sin ningún tipo de arrugas, en la ficha queiloscópica. 

### Análisis de la muestra

**Figura 1:**
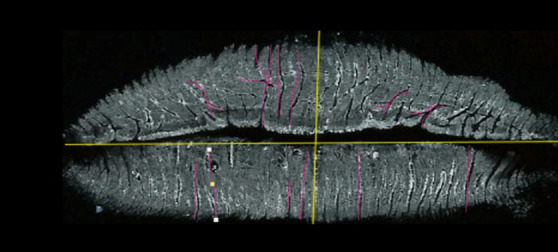
Imagen escaneada (caso 15/ escáner HP Deskjet F4283)

Una vez obtenidas las muestras, fueron escaneadas utilizando el escáner HP Deskjet F4283, ajustado a una resolución de 600 dpi. Para realizar el examen de las imágenes obtenidas, estas se transformaron a escala de grises. Las imágenes obtenidas se almacenan como Tagged Image File Format TIFF (formato de imágenes de alta resolución basado en etiquetas) en archivos con Adobe Photoshop 7.0 para el máximo de detalles (Figuras 1 y 2). 

**Figura 2:**
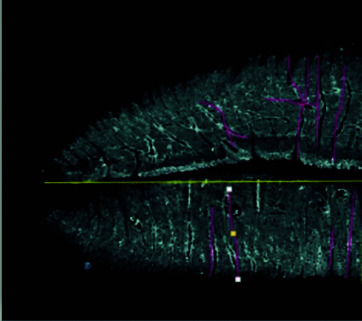
Imagen escaneada (caso 15/ escáner HP Deskjet F4283)

Se seleccionó cada impresión de labio de forma aleatoria y se amplió de manera uniforme en el ordenador hasta el 100% RGB, utilizando Adobe Photoshop 7.0 para el análisis. Luego, cada impresión del labio se analizó por cuadrante, con la finalidad de observar los tipos de surcos labiales que presentaba cada sujeto. 

La clasificación de los surcos expresados en las clases I a V se logró gracias a la propuesta por Suzuki y Tsuchihashi. Con los resultados de la interpretación de huellas labiales se logró determinar la identificación personal de cada sujeto.

La determinación del sexo se realizó posteriormente a la clasificación de las huellas labiales utilizando el método de Suzuki y Tsuchihashi, lo cual permitió aplicar los resultados dados por Vahanwala *et al*. con los patrones de labios más predominantes en cada cuadrante. 

Para finalizar la clasificación, se procedió a llenar el formulario de recojo de datos, que contiene la siguiente indagación: código, edad, género del alumno, grosor labial, disposición de comisuras, clasificación de Suzuki y Tsuchihashi y clasificación de Vahanwala *et al*., en las imágenes invertidas y por cuadrantes. 

Se calculó la longitud directa clínica y digital de los labios superior e inferior, con la regla ABO n.o 2. El cálculo de la longitud indirecta se realizó con la herramienta de medición (regla) del mencionado *software*. Para determinar el sexo, se utilizó el método propuesto por Vahanwala *et al*., que cuenta con patrones predominantes según el sexo del I al V. 

La base de datos incluyó cada una de las clasificaciones de los surcos labiales según el método, con el fin de facilitar la tabulación de los datos y su análisis estadístico posterior al vaciado de datos conseguidos a través de la tabla de recolección. 

La principal variable del estudio fue la determinación del sexo mediante la clasificación de los surcos labiales según la propuesta de Suzuki y Thuchihashi, que incluye la Clase I, líneas verticales completas; Clase I', líneas verticales incompletas; Clase II, líneas ramificadas o bifurcadas; Clase III, líneas entrecruzadas; Clase IV, líneas reticulares, y Clase V, líneas en otras formas.

A partir de la clasificación obtenida, se procedió a realizar la determinación del sexo utilizando el método de Vahanwala *et al*., utilizando los patrones predominantes en ambos labios, los cuales incluyen los diferentes patrones predominantes según sexo: Clase I y I', mujer; Clase I y II, mujer; Clase III, varón; Clase IV, varón, y Clase V, varón.

Se evaluó la distribución de frecuencias y porcentajes en cada análisis y, finalmente, la concordancia entre el resultado del método de Vahanwala *et al*. y la condición real del sexo fueron evaluadas con la prueba estadística de Kappa (p < 0,05).

## RESULTADOS

Se obtuvieron 94 huellas labiales, de las cuales 43 (46,8%) correspondían a sujetos de sexo masculino y 51 (53,2%), a sujetos de sexo femenino.

Se observó que no hubo una relación significativa entre el género y los extremos labiales. Según su disposición en proporción a la línea perpendicular media de los labios, presentaron mayor frecuencia para el tipo “horizontal” (ubicadas en la línea) tanto en el sexo masculino (67,4%) como en el femenino (47,1%) en la impresión labial, seguidas por las comisuras de tipo elevadas (25,5 %) y de tipo abatidas (19,11%) (Tabla 1).

**Tabla 1:** Frecuencia de la disposición de las comisuras según sexo

Disposición de las comisuras	Varonil		Femenina		Total		
	Frecuencias	Porcentajes	Frecuencias	Porcentajes	Frecuencias	Porcentaje
Abatidas	4	9,3	14	27,5	18	19,1
Horizontales	28	67,4	24	47,1	53	56,4
Elevadas	11	23,3	13	25,5	23	24,5
Total	43	100,0	51	100,0	94	100,0

Se observó que no existe una relación significativa entre el sexo y el grosor del labio. En los sujetos de sexo masculino, predominaron los labios de tipo mediano (46,5%), mientras que, para los sujetos de sexo femenino, predominó el tipo mixto (39,2%) (Tabla 2).

**Tabla 2:** Frecuencia del tipo de grosor del labio según sexo.

Tipo de grosor de labio	Varonil		Femenina		Total	
	Frecuencias	Porcentajes	Frecuencias	Porcentajes	Frecuencias	Porcentaje
Delgado	2	4,7	4	46,5	6	6,4
Mediana	20	46,5	16	31,4	36	38,3
Grueso	7	16,3	11	21,6	18	19,1
Mixto	13	30,2	20	39,2	33	35,1
Total	43	100,0	51	100,0	94	100,0

Se observó en la muestra la ausencia de una relación importante entre el sexo y la clase de surco del labio superior. En el caso del sexo masculino, los valores fueron los siguientes: clase I: 4,7%, clase I’: 2,3%, clase II: 11,6%, clase III: 7%, clase IV:51,2% y clase V: 23,3%; y para el sexo femenino: clase I: 11,8%, clase I’: 21,8%, clase II: 64,7%, clase III: 0%, clase IV: 2% y clase V: 0% (Tabla 3).

**Tabla 3:** Frecuencia de tipo de surco labial superior según sexo

Tipo de surcos en labio superior	Clasificación de Suzuki y Thsuchihashi					
	Varonil		Femenina		Total	
	Frecuencias	Porcentajes	Frecuencias	Porcentajes	Frecuencias	Porcentaje
Clase I	2	4,7	6	11,8	8	8,5
Clase I¨	1	2,3	11	21,6	12	12,8
Clase II	5	11,6	33	64,7	38	40,4
Clase III	3	7,0	0	,0	3	3,2
Clase IV	22	51,2	1	2,0	23	24,5
Clase V	10	23,3	0	,0	10	10,6
Total	43	100,0	51	100,0	94	100,0

Se observó que no existe una relación significativa entre género y la clase de surco del labio inferior. Para el sexo masculino los valores fueron los siguientes: clase I: 18,6%, clase I: 4,7%) clase II: 2,3%, clase III: 7,0%, clase IV: 41,9%, clase V: 25,6%; y para el sexo femenino: clase I: 82,4%, clase I’: 2%, clase II: 11,8%, clase III: 0%, clase IV: 2,0% y clase V: 0% (Tabla 4).

**Tabla 4:** Frecuencia de tipo de surco labial inferior según sexo

Tipo de surcos en labio inferior	Clasificación de Suzuki y Thsuchihashi					
	Varonil		Femenina		Total	
	Frecuencias	Porcentajes	Frecuencias	Porcentajes	Frecuencias	Porcentaje
Clase I	8	18,6	42	82,4	50	53,2
Clase I¨	2	4,7	1	2,0	3	3,2
Clase II	1	2,3	6	11,8	7	7,4
Clase III	3	7,0	0	,0	3	3,2
Clase IV	18	41,9	1	2,0	19	20,2
Clase V	11	25,6	1	2,0	12	12,8
Total	43	100,0	51	100,0	94	100,0

Sin embargo, se observó que sí existe una relación significativa entre el sexo y el patrón predominante en la muestra. En el caso del género masculino, tipos I y I’: 4,7%, clases I y II: 7,08%, clase III: 4,7%, clase IV: 58,1%, clase V: 25,6%; y, para el género femenino, clases I y I’: 21,68%, clases I y II: 74,5%, clase III: 0%, clase IV: 2% y clase V: 2% (Tabla 5).

**Tabla 5:** Concordancia entre el método Vahanwala *et al*. y el sexo real de la muestra estudiada

Método de Vahanwala et al.	Condición real del sexo		
	Masculino	Femenino	Total
Masculino	38	5	43
Femenino	2	49	51
Total	40	54	94

Kappa K = 0,849, p < 0,001

En el presente estudio, se observó que existe una relación significativa entre el sexo y el método Vahanwala *et al*. Se logró determinar el sexo de 38 varones y 49 mujeres fueron identificadas correctamente con base en los patrones predominantes en sus huellas labiales.

## DISCUSIÓN

Mantilla *et al*. ^12^ afirman que, en los últimos años, la odontología forense se ha transformado en herramienta científica de trascendencia para la medicina legal, debido a que, a lo largo de los años, se han desarrollado varios métodos que utilizan instrumentos de impresión de labios y fotos digitalizadas para determinar de manera confiable la identidad de una persona y para determinar el género y la raza. De igual modo, Téllez ^13^ señala que la identificación de huellas de labios ha demostrado ser una herramienta útil en criminología porque proporciona datos precisos y puede identificar a personas sospechosas al encontrar huellas de labios en la escena del crimen. Estudios realizados en el Perú sustentan que la queiloscopia es una tecnología de considerable valor científico para el reconocimiento, porque satisface los caracteres variable, inmutable, persistencia y categorización, al igual que la huella dactilar ^14^.

La presente investigación estuvo orientada al análisis de los cambios en los patrones y las características labiales en impresiones labiales para determinar el sexo en una muestra de 94 estudiantes, la cual estuvo compuesta por 44 hombres y 50 mujeres de la Institución Educativa Túpac Amaru del distrito de Chincheros, departamento de Apurímac. Las edades fueron de 15 a 18 años, ciudadanía peruana y no hubi distinción de género o raza. Se determinó que el 88,7% de los estudiantes fue identificado correctamente como sexo masculino y el 11,3% no se identificó por presentar patrones de tipo II t tipo II’; sin embargo, el 92% de estudiantes se logró identificar plenamente como sexo femenino y el 3% de estudiantes no se logró clasificar por presentar patrones que no se encontraban en la propuesta de clasificación de Vahanwala *et al*. ^15^, las cuales fueron tipo II y I’. Solo un estudiante no se logró determinar por presentar patrón de tipo V.

Moya *et al*. ^16^ muestran que los cambios y características de la forma del labio deben sumarse al análisis queiloscópico. De igual forma. Cuesta *et al*. ^17^ destacan las principales características de la investigación en cuanto al grueso, distribución de comisura e impresión labial, las cuales fueron consideradas durante la investigación y analizadas en esta investigación.

Con relación a las comisuras labiales. Cuesta *et al*. ^17^ compararon la topografía de los labios del grupo familiar y demostraron que el tipo de comisuras labiales abatido y horizontal fueron los más frecuentes. Mientras que Rodríguez ^14^, en estudio que realizó en una población peruana para determinar el sexo mediante la inspección de labios, la marca de los labios y la foto digitalizada, demostró que la clase de comisuras labiales abatidas fueron las más frecuentes para ambos sexos. Sin embargo, para la presente investigación las comisuras de tipo horizontal y elevada fueron las más frecuentes. Esta divergencia en los resultados podría deberse a la diferencia que existe entre los grupos de población estudiados y los tipos de geografía en los cuales se realizaron los estudios. En el trabajo realizado por Cuesta *et al*. ^17^, la población estudiada fue de grupos familiares conformados por padre, madre e hijos biológicos en una población de Colombia. En el estudio realizado por Rodríguez ^15^, la población fueron estudiantes peruanos de la región Lima sin parentesco familiar alguno, mientras que en el actual trabajo la población estudiada fueron estudiantes peruanos de la región Apurímac. Se puede ver que no hay diferencia significativa entre el sexo y los extremos labiales.

Sobre el grueso de los labios y su relación directa con el reconocimiento étnico, Cuesta *et al*. ^17^ y Negre ^18^ aplicaron la clasificación diseñada por Moya *et al*. ^19^ y la utilizaron como referencia para el examen del grosor de los labios. Señalan que el tipo de labio delgado se registró con mayor frecuencia, seguido por el tipo de labios gruesos, medianos y, los restantes, mixtos. No se observan coincidencias totales en labio superior. Con respecto al grosor del labio, en el estudio realizado por Rodríguez ^14^ se logró clasificar el mismo tipo tanto en la impresión como en la fotografía, siendo más frecuente el tipo de labios medianos para el género masculino y para el género femenino. En la presente investigación, los resultados indican que el tipo de labios mixtos se presentó con mayor frecuencia, seguido por el tipo de labios mixtos, gruesos y, por último, delgados. La diferencia en los resultados podría deberse a la diferencia que existe entre los grupos de poblaciones estudiadas y los tipos de geografías. Se observa que no existe diferencia significativa entre sexo y grosor de labios.

Con relación a las estampas labiales, Tsuchihashi ^20^, en su estudio, en el que participaron 1364 sujetos japoneses e incluyó niños y adultos, concluyó que las huellas de labios analizadas no muestran patrones similares. y cada uno es único. Asimismo, Mantilla *et al*. ^12^ analizaron las impresiones labiales de 60 estudiantes de la Universidad Tecnológica de Santander en Colombia y el resultado fue que las impresiones labiales de cada individuo demostraron su personalidad y fueron consistentes con los resultados de esta investigación.

Otros trabajos verificaron la efectividad de las huellas de labios como herramienta de investigación forense y para probar su eficacia en la determinación de género, concluyendo que el uso del examen de la identificación odontología como técnica es una herramienta útil ^21^^-^^25^.

Ashish *et al*. ^19^ realizaron estudios con el objetivo de determinar la distribución de los patrones de labios predominantes y así evaluar si existen diferencias entre ambos sexos en una población de la India. Para el estudio se seleccionó a 400 adultos entre mujeres y varones. De acuerdo con la categorización de Suzuki y Tsuchihashi, la clase IV y la clase III fueron los patrones más y menos prevalentes, respectivamente. Se encontró que el patrón de labios tipo II era el más predominante en los varones, mientras que la clase IV se encontró con mayor frecuencia en las mujeres. La presente investigación difiere de los resultados obtenidos por Aishish *et al*. ^19^ ya que, para el presente estudio, el modelo preponderante en el género masculino fue la clase IV, mientras que, en el género femenino, los patrones predominantes fueron las clases I y II. La diferencia podría ser resultado de la diferencia entre las poblaciones estudiadas y los tipos de geografía en los cuales se realizaron los estudios.

Los resultados de la investigación realizada por Ballur *et al.*^26^, con respecto al análisis de surcos labiales, obtuvo como resultado que el tipo I fue el más común en todos los sujetos. El patrón de tipo III fue el que predominó en el sexo femenino. Las clases I, I’ y II predominaron en las mujeres, mientras que las clases III, IV y V predominaron en los hombres. En la presente investigación, los resultados coinciden con los obtenidos, los cuales determinaron que la clase I fue la más común en todos los estudiantes; las clases IV, V y III predominaron en el sexo femenino y las clases I, I’ y II, en el sexo masculino. 

Los estudios realizados por Verma *et al*. ^27^ demostraron que los patrones de impresiones de labios que se obtenían de forma directa e indirecta, tanto en mujeres como en varones de diferentes edades, no varía con el tiempo. El estudio se realizó efectuando el proceso de inter e intraexaminador, durante un periodo de 6 meses en los que no se observó ningún cambio en la población estudiada, que constó de 100 sujetos (50 mujeres y 50 hombres, con edades entre 15 y 35 años). Como resultado del estudio, se encontró que los patrones predominantes eran verticales y ramificados. Las mujeres mostraron el patrón ramificado y los varones revelaron una prevalencia igual de patrones verticales y reticulares; lo cual demuestra que las huellas labiales pueden ser utilizadas como una herramienta forense fiable. 

Estos resultados concuerdan con los obtenidos por Verma *et al*. ^27^, quienes hallaron que los patrones predominantes fueron los verticales y ramificadas. Para el sexo femenino mostraron el patrón ramificado y los varones revelaron una prevalencia igual de patrones verticales y reticulares.

La investigación realizada por Rodríguez ^14^ utilizando el método propuesto por Vahanwala *et al*., comprobó la precisión de la queiloscopia para la determinación del género en las impresiones de labios y fotografías digitales de estudiantes graduados de la Universidad Científica del Sur. El patrón predomínate para el género femenino fueron los tipos I y IV y, para el género masculino, la clases V y IV. La reciente investigación difiere de los resultados obtenidos por Rodríguez, al ser más frecuente el patrón predominante propuesto por Vahanwala et al. para el género femenino las clases I y II, y para el género masculino, la clase IV. La diferencia en los resultados podría deberse a la diferencia que existe entre los grupos de población estudiados y los tipos de geografías en los cuales se realizaron los estudios.

En relación con los resultados obtenidos luego del análisis de las impresiones labiales y fotografías digitales -métodos escogidos para realizar el análisis de las huellas labiales-, estos no mostraron diferencias estadísticamente significativas en los estudios de grosor labial utilizados para determinar la raza y la comisura labial. Cabe señalar que se encontraron diferencias importantes en el análisis del pliegue labial, pues las fotos digitales fueron observadas con mayor precisión.

Por otro lado. se debe considerar que el tamaño muestra de esta investigación no es grande, por lo que nuestros resultados no pueden generalizarse a toda la población peruana. Por tal motivo, se deben corroborar estos resultados y hallazgos con estudios posteriores. Finalmente, la identificación del sexo con el método de Vahanwala *et al*. mostró tener una eficacia cercana al 90% para ambos sexos, por lo que su utilidad en el ámbito forense y académico es importante.

## CONCLUSIONES

La queiloscopia como herramienta para la determinación del sexo en el ámbito forense parece ser un método útil, ya que permite encontrar diferencias marcadas entre ambos sexos, lo cual ayuda a distinguirlos a partir de sus huellas labiales. 

## Fuente de financiamiento:

Autofinanciado
